# A choice experiment assessment of stated early response to COVID-19 vaccines in the USA

**DOI:** 10.1186/s13561-022-00368-w

**Published:** 2022-03-31

**Authors:** Ricardo A. Daziano

**Affiliations:** School of Civil and Environmental Engineering, Cornell University, Ithaca, 14853 NY USA

**Keywords:** Choice experiment, Vaccine, COVID-19, Conditional logit

## Abstract

**Background:**

Using choice microdata (N=2723) across the USA, this paper analyzes elicited acceptance of hypothetical COVID-19 vaccines.

**Methods:**

The hypothetical vaccines in a choice experiment were described in terms of effectiveness, days for antibodies to develop, duration of protection, risk of both mild and severe side effects, which health agency mainly supports the vaccine, country of origin, and when the vaccine was developed. Out-of-pocket cost was also considered as characteristic of the vaccines to derive welfare measures.

**Results:**

All vaccine attributes had expected signs with significant estimates. Vaccines developed in the USA and the UK were preferred to a hypothetical German vaccine, whereas a Chinese origin was very negatively perceived. Since the choice scenarios also gave the option to opt out from taking the vaccine, odds ratios were derived to characterize the segments that are more and less likely to accept vaccination. More likely to opt out were found to be those who stated to be against vaccination in general, African Americans, individuals without health insurance, and older people. Males, democrats, those who took the flu vaccine appear as more willing to accept vaccination.

**Conclusions:**

Estimates of the fitted choice models in this study are informative for current and future immunization programs.

## Introduction

Multiple international efforts have been working at an unprecedented pace to make the coronavirus disease 2019 (caused by the severe acute respiratory syndrome coronavirus 2 SARS-CoV-2), a vaccine-preventable disease. Success of immunization programs aiming at stopping the COVID-19 pandemic will depend on acceptance rates of vaccination. These acceptance rates of mass immunization are a function of both characteristics or attributes of the available vaccines and individuals’ attitudes toward vaccination.

Vaccines need to be both effective and safe. On the one hand, effectiveness of a vaccine is measured as the expected percent reduction in the frequency of illness among vaccinated people compared to those not vaccinated. Effectiveness of vaccines varies, as does duration of protection against the target disease. For example, according to information provided by both the Centers for Disease Control and Prevention in the USA (CDC) and the World Health Organization (WHO), the vaccine against measles is 97% effective with protection that lasts over 20 years with 2 doses (90% effective with only 1 dose); other examples include yellow fever (99% effective after 30 days of immunization, protection >30 years, 1 dose), hepatitis A (99%, >20 years, 2 doses), TB (50%, >20 years, 1 dose), and chickenpox (90-95%, >20 years, 1 dose). In the case of the seasonal flu, the vaccine is only 40-60% effective (due to the multiple strains that are present each flu season). To approve a vaccine against COVID-19, the Food and Drug Administration in the USA (FDA) established a lower bound of 50% for effectiveness (June 2020).

On the other hand, safety of a vaccine is related to complications or side effects. Whereas individuals receiving a vaccine may experience mild symptoms such as temporary nausea, rash, fever, and body aches (often as soreness of the muscle where the injection was given), complications can be severe. For example, it has been shown that fewer than 1 or 2 cases per million of Guillain-Barré syndrome can be associated with the flu vaccine.

Pivoting around effectiveness and safety, this study seeks to inform immunization programs by analyzing elicited acceptance of a vaccine to prevent further spread of COVID-19 using choice experiments (cf. [1],). International efforts for a COVID-19 vaccine by November 2020, when data for this study was collected, counted a total of 54 vaccines under clinical trials. On November 9 – 2 weeks and a half after this study started field work, scientists of the effort led by the New York-based company Pfizer and the German company BioNTech announced that their vaccines had over 90% efficacy. On November 16, the American biotechnology company Moderna followed with an announcement of their vaccine being 94.5% effective. On November 23, Swedish-British AstraZeneca announced their vaccine, developed with the University of Oxford, was 70.4% effective on average (when more than 1 dose was used in their clinical trials, efficacy was above 90%).

The rest of the paper is organized as follows. Literature review section summarizes related, previous research that has exploited choice experiments to analyze response to vaccination. Methods section discusses the methods, including description of the survey and choice experiment, microdata, and general specification of the adopted choice models. Results section presents and discusses the results of a series of choice models in the logit family, namely conditional logit with observed heterogeneity, conditional logit with a random effect that accounts for multiple choices by the same individual, and latent class conditional logit specifications. Preference point estimates as well as analysis of willingness-to-pay metrics and inference on odds ratios of accepting and rejecting vaccination are analyzed. Discussion and conclusions section concludes.

## Literature review

Choice experiments (CEs) are a common tool in health economics [2, 3] to determine valuation of treatments, including response to vaccination (see [4],for a recent review of studies focused on health).

For example, [5] used microdata collected in an online survey in the Netherlands (N=536) to examine preferences toward vaccination in the case of a pandemic outbreak. The choice experiment included the following vaccine attributes: effectiveness, safety, advice, media coverage, and out-of-pocket costs. In addition to the attributes of the alternative vaccines, the experiment described the pandemic outbreak in terms of two attributes, namely: susceptibility and severity of the disease. Using a latent class logit model, the study found a willingness to pay in the order of €6-39 for a 10% increase in effectiveness of the vaccine, depending on severity of the pandemic. [6] report estimates of the same choice experiment for samples of respondents in Poland (N=510), Spain (N=512), and Sweden (N=510).

Focusing on decisions regarding seasonal influenza vaccination programs made by physicians in Hong Kong, [1] implemented a choice experiment in a survey (N=258) that was administered both in person and online. Attributes of the hypothetical influenza vaccines included: efficacy and probability of mild adverse effects. Program characteristics were also considered as experimental conditions, namely: duration in months, vaccination location, arrangement procedure (appointments vs. walkins), and service hours. Finally, the proportion of healthcare professionals intending to receive the influenza vaccine was also included as proxy for social norms. By analyzing compensatory effects, the authors concluded that the probability of side effects was weighed higher than efficacy.

Another recent study relevant to ours is that of [7], where the authors discuss external validity of predictions based on responses to choice experiments and conclude that correct aggregate predictions are attained when preference heterogeneity is taken into account. Participants of the study in the Netherlands (N=1200) were randomized to one of six choice experiments. The first of the two medical conditions under analysis was influenza vaccination, and the second condition was colorectal cancer screening (CCS). In the case of influenza vaccine, the authors used the vaccine attributes of the choice experiment described in [8], namely: effectiveness, risk of severe side effects (as number of people out of 1,000,000), risk of mild side effects (as number of people out of 10), protection duration, and number of weeks for vaccine to become active. The attributes for the CCS choice experiment in [7] were: effectiveness, probability that the screening test does not find the disease in people who are ill (false negative test), waiting time for test results, waiting time for follow up, and frequency of screening.

## Methods

### Data

**Fig. 1:**
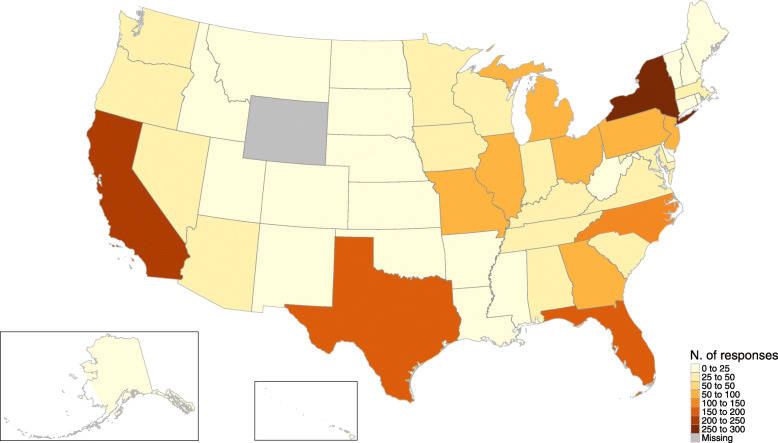
Responses to the online survey by state

The data (N=2723) were collected from October 22 of 2020 to November 24 of 2020 using an online panel, managed by Qualtrics, of adults across the United States (Fig. 1). It is worth mentioning that the last 1,049 responses were collected right after the announcements by Pfizer (November 9), Moderna (November 16), and AstraZeneca-Oxford (November 23) about efficacy of their vaccines. Given this unplanned external shock, these observations were labelled as Wave 2 of the on-going survey. During the first wave of data collection, little was known about what would be the characteristics of a vaccine. Table 1 summarizes characteristics of the full sample and Table 2 focuses on answers to health-related questions. As working definition of age generations we adopted cutoffs that are standard in the US, namely: millennials (born between 1981 and 1996), Generation X (1965-1980), and baby boomers (1946-1964).

**Table 1:** Sample Demographic Statistics

Variable	% of sample
Male	44.95%
Married	49.91%
Race: White	76.17%
Race: Black or African American	13.51%
Race: Asian or Asian American	4.19%
Ethnicity: Hispanic (of any race)	12.45%
Full-time worker	48.37%
Part-time worker	6.35%
Self-employed	7.57%
Working or studying from home	35.11%
Retired	12.30%
Student	3.31%
Bachelor’s degree	22.29%
Master’s degree	15.28%
Doctoral degree	2.46%
Millennial	49.50%
Generation X	28.68%
Baby boomer	19.24%
Older than baby boomer	2.57%
*Political views*	
Democrat	42.09%
Republican	29.93%
Independent	22.07%
*Division*	
East North Central	13.48%
East South Central	6.50%
Middle Atlantic	18.95%
Mountain	5.55%
New England	3.89%
Pacific	11.97%
South Atlantic	23.50%
West North Central	6.54%
West South Central	9.62%
*Household income*	
Less than $40,000	39.09%
$40,000-75,000	27.88%
$75,000-100,000	11.24%
$100,000-125,000	6.36%
$125,000-150,000	5.03%
$150,000-200,000	5.18%
$200,000 or more	5.21%

**Table 2:** Sample Health-Related Characteristics

Variable	% of sample
Vaccinated against flu winter 20/21	34.52%
Against vaccination in general	5.40%
*COVID-19*	
Have been tested	22.33%
Have been tested positive	4.11%
*Self-assessed Health Status*	
Excellent	20.64%
Good	42.05%
Average	26.96%
Poor	8.67%
Very poor	1.69%
*Health Insurance*	
Through work	24.54%
Covered by spouse’s or family member’s	13.97%
Governmental insurance	37.28%
No insurance	13.56%

The online survey contained several sections, including self-assessment of health status, stated behavioral changes and preventive actions in response to the pandemic, attitudes toward vaccination, and choice experiments with controlled hypothetical scenarios of vaccination and testing alternatives.

Table 3 compares selected characteristics of the sample, disaggregated by wave, with figures from the US 2020 census. Our sample is younger, more educated and yet with a lower median income than the US population. Even though our sample has a lower representation of Hispanics, in terms of race the composition of our sample is similar to that of the population. Our sample has slightly fewer males.

**Table 3:** Comparison of sample sociodemograhics with census data

Variable	US Census	Wave 1	Wave 2
Median age [years]	49	39	39
People aged 65+ [%]	21.2%	10.9%	9.4%
Male [%]	49.2%	46.7%	39.8%
Race: White [%]	76.3%	70.3%	78.6%
Race: Black or African American [%]	13.4%	17.5%	11.2%
Race: Asian or Asian American [%]	5.9%	4.8%	4.6%
Ethnicity: Hispanic of any race [%]	18.5%	15.2%	10.0%
Median household income [US$]	$62,843	$45,000	$45,000
No health insurance [%]	10.2%	13.1%	15.2%
Education: High school graduate [%]	88.0%	96.0%	97.4%
Education: Bachelor’s degree or higher [%]	32.1%	38.2%	37.9%

### Discrete choice experiment

In this study, a choice experiment was constructed around the availability of hypothetical COVID-19 vaccines. The design and initial roll out of the choice experiment were performed before any official announcement of actual vaccines and their clinical studies was made. Each hypothetical vaccine in the choice experiment was described in terms of attributes with levels that were pivoted around known characteristics of the vaccine against the flu, such as: effectiveness, immune response, duration of protection, and risk of side effects (cf. [8],). The experiment was also designed to consider who mainly supports or suggests [5] a given vaccine (CDC versus WHO, for example), and for how long the vaccine has been used as a proxy for dependability. Out-of-pocket costs were included as attribute to ensure the possibility of deriving welfare estimates [9]; however, the possibility of the vaccine being free was also included. Finally, country of origin of the vaccine was another vaccine characteristic. These attributes were chosen following characteristics of the vaccine against the seasonal flu, findings from the literature review, and outcomes from an online focus group. Characteristics of actual vaccines were not known at the time of the first wave of data collection (late October of 2020). Some features, such as duration of protection, remained unknown to scientists and the public even at the end of rollout of the survey.

After an online focus group and pretest, a Bayesian efficient design of the choice experiment was created with the attributes and levels shown in Table 4. A total of 24 choice scenarios were generated in the design, each respondent was assigned to a random subset of 7 choice cards.

**Table 4:** Vaccine CE: experimental attributes and levels

Attribute	Levels
Out-of-pocket cost [$]	$0, $50, $100, $175
Effectiveness [%]	20%, 40%, 60%, 80%
Days for antibodies to develop	7, 14, 21 days
Duration of protection [months]	3, 6, 12 months
Number of people out of 10 with mild side effects	1, 3, 5 out of 10
Number of people out of 1,000,000 with severe side effects	1, 10, 100 out of 1,000,000
Who recommends this specific vaccine	PCP (base), CDC, WHO, Media
Country where vaccine was developed	Germany (base), USA, UK, China
When vaccine was developed [months]	3, 6 months

The same units of the study by [8] were adopted for effectiveness, risk of both mild and severe complications, and duration of protection. However, based on our focus group, waiting time for the vaccine to produce enough antibodies was presented as days (cf. weeks in [8],). When designing the choice experiment, it was not known yet that two doses with a 21-28 day waiting time between each dose would be required for the vaccines. Mild side effects were presented as temporary pain at the site of injection, headache, and fatigue that would not need any special care, whereas severe side effects were presented as a systemic adverse reaction that would require medical care, giving a severe allergic reaction as example. Regarding the actual levels for effectiveness (20-80%), it is important to note that whereas the FDA established lower bound of 50% for effectiveness in June 2020, the media at the time of unknown effectiveness of COVID-19 vaccines heavily reported that flu vaccines usually range from 40 to 60%, but could be as low as 10% effective as in the 2004/5 season. Actual effectiveness reaching 90% or higher as announced toward the end of 2020 came as an unexpected high.

**Fig. 2:**
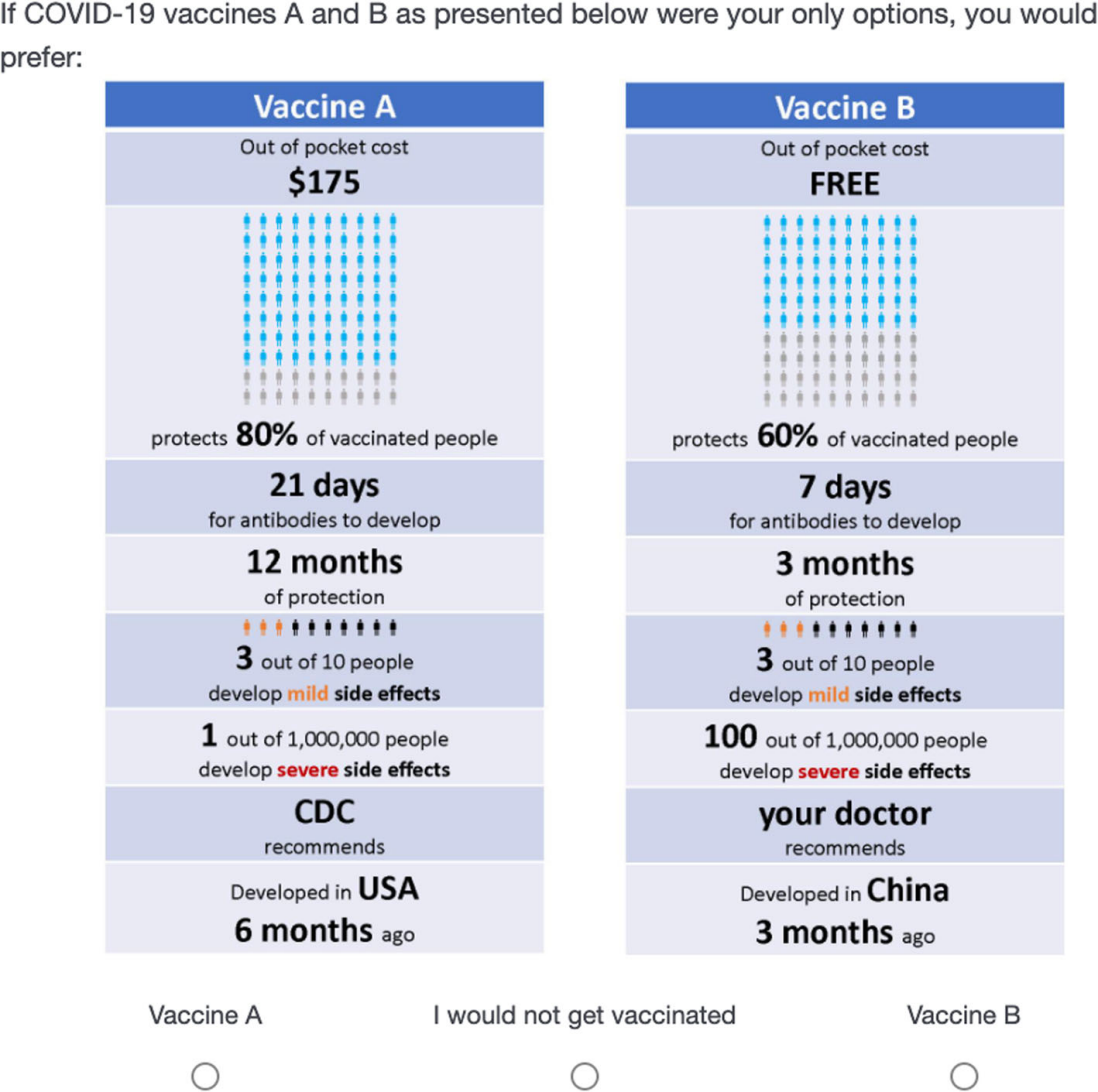
Vaccine CE: sample choice card

As in [5], we included who is backing up a specific vaccine. Unlike the cited study – that considered ‘media coverage’ as an independent attribute from what they called ‘advice’, we used ‘media’ as an additional level of who is mostly recommending the vaccine in question. In the models, the primary care physician (PCP, presented as “your doctor”) in the choice cards (Fig. 2) was set as base or reference level.

Regarding development of the vaccine, the timeline was presented to respondents as the number of months since successful clinical trials. To give context to this attribute, the Moderna vaccine was first actually administered to a volunteer in Seattle in March 2020, whereas emergency use in the US was authorized in December of the same year (9 months). Phase III clinical trials started by the end of July 2020 (slightly less than 5 months prior to emergency use authorization). Scientists mentioned at the time that future developments for emerging strains could take as little as six weeks.

### Conditional logit specification

Consider a standard discrete choice setup [10] with the possibility of opting out (*o*), where individual *i* chooses among *J* alternatives over *T* time periods (the choice set being $\mathcal {C}=\{o,1,\dots,J\}$), and that the truncated indirect utility from alternative *j* in period *t* is:

1 $$\begin{array}{*{20}l} u_{ijt} &= \mathbf{x}_{ijt}' (\boldsymbol{\beta} + \boldsymbol{\Pi} \mathbf{w}_{i}) + \varepsilon_{ijt}  \end{array} $$

2 $$\begin{array}{*{20}l} u_{iot} &= \delta_{io} + \mathbf{w}_{i}'\boldsymbol{\gamma} + \varepsilon_{iot},  \end{array} $$

where **x**_*ijt*_ is a vector of choice-specific attributes, ***β*** is a vector of population mean preferences, ***Π*** is a parameter matrix representing observed preference heterogeneity with respect to the population mean ***β*** that are interacted with individual-specific characteristics **w**_*i*_,*δ*_*io*_ is a constant for the opt-out option *o*, ***γ*** is a parameter vector identifying how sociodemographics makes an individual more or less likely to opt out from choosing an alternative, and *ε*_*i*·*t*_ is an iid type-I extreme value preference shock (leading to a logit kernel). If the opt-out constant is fixed, i.e., *δ*_*o*_, the underlying model is a conditional logit [11]. The conditional logit model, in addition to restrict substitution patterns across alternatives to being constant, treats each observation *t* as a pseudo-individual. In other words, the conditional logit model neglects the panel structure of the data that comes from an individual stating a response to multiple choice situations.

To deal with the panel nature of data with multiple observations (choice situations *T*), we further assume that *δ*_*io*_ is random and normally distributed, i.e., $\delta _{io}\sim \mathcal {N}(\delta _{o},\sigma _{o}^{2})$. The normally distributed opt-out constant is a random effect that leads to a random parameter logit [12]. Note that in addition to account for unobserved preference heterogeneity, having a random *δ*_*io*_ also makes the total variance of the opt-out alternative to be different than the variance of the random utility of each of the two vaccines. Note that in terms of covariance structure, our random parameter logit specification is equivalent to a nested logit model [9, 13] with a single-alternative nest.

Whereas Eq. 1 clearly specifies a truncated indirect utility function of a conditional logit model, Eq. 2 specifies an index function of a multinomial logit model. In this sense, the parameter vector ***β*** and the parameter matrix ***Π*** contain marginal utilities that can be used to derive estimates of marginal rates of substitution. In particular, if *β*_cost_ is the marginal disutility of cost (which equals the additive inverse of the marginal utility of income), then the parameter ratio in Eq.3 represents the maximum willingness to pay for a marginal improvement in attribute *k* of alternative *j*:

3 $$ \omega_{k}=-\frac{\partial u_{ijt}/\partial x_{kijt}}{\partial u_{ijt}/\partial \text{cost}_{kijt}}=-\frac{\beta_{k}}{\beta_{\text{cost}}}.   $$

In the case of observed preference heterogeneity, say between attribute *k* and individual characteristic *l*, the maximum willingness to pay simply becomes *ω*_*kl*_=−(*β*_*k*_+*π*_*kl*_w_*l*_)/*β*_cost_.

On the other hand, the parameter vector ***γ*** can be interpreted as a vector of odds ratios between opting out and opting in when the transformation OR= exp(***γ***) is used. These estimates are particularly relevant for characterizing the segments of the population that are more or less likely to take a vaccine.

Finally, whereas the maximum likelihood estimator can be used for the conditional logit model, the random parameter logit specification needs implementation of the maximum simulated likelihood estimator [12].

### Latent class conditional logit specification

In addition to a single random effect, the choice model in Equations 1 and 2 can be specified as a latent class conditional logit model LCL [14–16] if a discrete heterogeneity distribution is assumed for all parameters in the model, i.e.

4 $$\begin{array}{@{}rcl@{}} u_{ijt} &=& \mathbf{x}_{ijt}' (\boldsymbol{\beta}_{i} + \boldsymbol{\Pi}_{i} \mathbf{w}_{i}) + \varepsilon_{ijt}  \end{array} $$

5 $$\begin{array}{@{}rcl@{}} u_{iot} &=& \delta_{io} + \varepsilon_{iot},  \end{array} $$

where the preference parameters ***β***_*i*_ and those in the matrix ***Π***_*i*_ along with the opt-out constant *δ*_*io*_ follow a discrete distribution with probability given by a multinomial logit specification. If the vector ***θ***_*i*_ recovers all these parameters, then the probability that the random ***θ***_*i*_ takes the specific value ***θ***_*q*_ – among *Q* possible values – is given by [16]:

6 $$ \Pr(\boldsymbol{\theta}_{i}=\boldsymbol{\theta}_{q})=\pi_{iq} =\frac{\exp(\gamma_{q}+ \mathbf{w}_{i}'\boldsymbol{\gamma}_{q})}{\sum_{q=1}^{Q} \exp(\gamma_{q}+ \mathbf{w}_{i}'\boldsymbol{\gamma}_{q})}.  $$

Note that in this LCL specification the sociodemographics **w**_*i*_ inform assignment to classes as a function of the parameters ***γ***. In practice, the number of classes *Q* is set before estimation of the model. Even though the LCL model has a straightforward derivation, its loglikelihood is not concave and implementation of the maximum likelihood estimator can face convergence problems. A partial solution is the use of the iterative Expectation-Maximization (EM) algorithm [17].

## Results

### Conditional logit: preference parameters

Respondents traded off between the two hypotehtical vaccines presented on each choice card, and chose to take one of the vaccines in 71.67% of the scenarios. After preliminary analyses to identify an informative specification with overall acceptable significance of the covariates, Table 5 presents the point estimates of the preference parameters of both the conditional logit (Cond. logit, with all parameters being nonrandom) and the logit model with a random effect (RPL), together with stars indicating statistical significance of a two-tailed test. A linear specification for cost (1) and a nonlinear specification (2) where the vaccine being free had its own parameter (*β*_Free_) were implemented. Based on the Bayesian Information Criterion (BIC), the random parameter logit model accounting for a separate preference parameter for the vaccine being free (i.e, model RPL 2 in Table 5) is preferred.

**Table 5:** Point estimates

Variable	Cond. logit 1	RPL 1	Cond. logit 2	RPL 2
*Preferences:* ***β***				
Free	NA	NA	0.4116***	0.5126***
Cost [$]	-0.0043***	-0.0047***	-0.0030***	-0.0030***
Effectiveness [%]	0.0102***	0.0111***	0.0110***	0.0122***
Protection [months]	0.0311***	0.0319***	0.0319***	0.0331***
Incubation [days]	-0.0120***	-0.0111***	-0.0078***	-0.0056*
Severe side effects [out of 10^6^]	-0.0024***	-0.0027***	-0.0026***	-0.0029***
Mild side effects [out of 10]	-0.0355***	-0.0515***	-0.0486***	-0.0623***
Introduced [months]	0.0340***	0.0282***	0.0497***	0.0459***
*Origin (base: Germany)*				
USA	0.2687***	0.4327***	0.3105***	0.4721***
UK	0.1587**	0.2527***	0.1900***	0.3350***
China	-0.3132***	-0.3046***	-0.1938***	-0.1291**
*Recommends (base: PCP)*				
Media	-0.3864***	-0.5599***	-0.4618***	-0.6660***
CDC	-0.0286	-0.0415	-0.0471	-0.1476**
WHO	-0.0648	-0.2166***	-0.1401***	-0.3281***
*Heterogeneity:* ***Π***				
Cost × Income ≥ $120K	0.0024***	0.0024***	0.0024***	0.0024***
Effectiveness × Wave 2	0.0030***	0.0022***	0.0030***	0.0022***
China × Republican	-0.1629***	-0.2017***	-0.1628***	-0.2051***
*Opt-out:* ***γ***				
Opt-out constant (mean)	1.0540***	0.6720***	0.7983***	0.3572*
Opt-out constant (st.dev.)		3.2605***		3.2693***
Male	-0.6604***	-1.4286***	-0.6611***	-1.4326***
Household Income [$10K]	-0.0172***	-0.0557***	-0.0173***	-0.0559***
Education BSc	-0.1492***	-0.3383***	-0.1467***	-0.3345***
Education Postgraduate	-0.6764***	-1.5117***	-0.6765***	-1.5143***
Age	0.0257***	0.0571***	0.0257***	0.0573***
Black or African American	0.3006***	0.7639***	0.3026***	0.7665***
Hispanic	-0.0123	0.0445	-0.0103	0.0462
Asian or Asian American	0.0833	0.3388**	0.0797	0.3339**
Religion extremely important	-0.1637***	-0.4939***	-0.1631***	-0.4938***
Democrat	-0.5340***	-1.1452***	-0.5356***	-1.1516***
Tested for COVID-19	-0.1395***	-0.3627***	-0.1394***	-0.3637***
Had COVID-19	-0.3353***	-0.2013	-0.3394***	-0.2049
No health insurance	0.1962***	0.5062***	0.1989***	0.5111***
Had flu shot	-0.5152***	-1.0224***	-0.5164***	-1.0262***
Against vaccination	1.7706***	4.1637***	1.7707***	4.1708***
Older than 65 in household	-0.2380***	-0.4946***	-0.2380***	-0.4951***
BMI	-0.0095***	-0.0200***	-0.0095***	-0.0200***
Underlying condition	-0.3240***	-0.6834***	-0.3229***	-0.6832***
Works full time	-0.4177***	-0.9344***	-0.4197***	-0.9382***
Loglikelihood	-18425.00	-15219.00	-18385.00	-15219.00
BIC	37205.10	30802.81	37134.60	30714.34

Note: Significance codes ^.^*p*<0.1,^∗^*p*<0.05,∗∗*p*<0.01,∗∗∗*p*<0.001

From the signs of the statistically significant marginal utilities ***β*** and as expected, individuals prefer a vaccine with higher effectiveness, longer protection, and that has been available for a longer period of time. Individuals also showed preference for vaccines coming from the USA or the UK as compared to a German origin. Vaccines with a longer waiting time for enough antibodies to develop, higher risk of both mild and severe side effects, and coming from China are less preferred. In general, the point estimates and their significance of the conditional and random parameter logit models are comparable. Note that whereas the World Health Organization WHO being the main health agency recommending a vaccine was not significant for the conditional logit 1, when either allowing the opt-out constant to be random or when introducing *β*_Free_, the WHO is associated with a significant and negative (with respect to the PCP of the respondent) point estimate. The magnitude of preference over the vaccine attributes is better illustrated by looking at their economic valution, as discussed in the next subsection.

### Conditional logit: willingness to pay

From the preferences parameters, estimates of the maximum willingness to pay (WTP) for a marginal improvement in the attributes of the vaccines were derived. These estimates are shown in Table 6. Because of the statistically significant interaction of the marginal utility of income with household income above US$ 120,000 (Cost × Income ≥ $120K), the WTP estimates are presented for two income groups.

**Table 6:** Maximum marginal willingness-to-pay (WTP) estimates

Variable	Cond. logit 1	RPL 1	Cond. logit 2	RPL 2
*Household income < $120K*				
Effectiveness [%]	$2.40	$2.38	$3.70	$4.09
Effectiveness × Wave 2	$3.11	$2.85	$4.71	$4.81
Protection [months]	$7.32	$6.83	$10.70	$11.03
Incubation [days]	-$2.82	-$2.28	-$2.60	-$1.87
Severe side effects [out of 10^6^]	-$0.57	-$0.57	-$0.87	-$0.97
Mild side effects [out of 10]	-$8.34	-$11.02	-$16.31	-$20.79
Introduced [months]	$7.98	$6.04	$16.67	$15.32
*Origin (base: Germany)*				
USA	$63.17	$92.64	$104.18	$157.48
UK	$37.31	$54.1	$63.76	$111.76
China	-$73.63	-$65.22	-$65.02	-$43.05
China × Republican	-$111.90	-$108.40	-$119.64	-$111.47
*Recommends (base: PCP)*				
Media	-$90.83	-$119.87	-$154.94	-$222.19
CDC				-$49.23
WHO	-$15.22	-$46.37	-$47.02	-$109.45
*Household income ≥ $120K*				
Effectiveness [%]	$5.49	$4.89	$18.54	$20.25
Effectiveness × Wave 2	$7.11	$5.86	$23.62	$23.86
Protection [months]	$16.74	$14.02	$83.60	$54.69
Incubation [days]	-$6.45	-$4.89	-$13.05	-$9.25
Severe side effects [out of 10^6^]	-$1.21	-$1.18	-$4.39	-$4.81
Mild side effects [out of 10]	-$19.08	-$22.64	-$81.81	-$103.04
Introduced [months]	$18.26	$12.41	$83.60	$75.96
*Origin (base: Germany)*				
USA	$144.55	$190.33	$522.47	$780.61
UK	$85.39	$111.15	$319.77	$553.95
China	-$168.49	-$133.99	-$326.09	-$213.40
China × Republican	-$256.09	-$222.7	-$599.98	-$552.53
*Recommends (base: PCP)*				
Media	-$207.86	-$246.26	-$777.00	-$1101.34
CDC				-$244.01
WHO	-$34.84	-$95.27	-$235.80	-$542.51

For the first wave, the maximum willingness to pay for a 10% increase in effectiveness of the vaccine is roughly $24-$40, depending on the model, for individuals in the lower income group and $49-$200 for the higher income group. For the second wave, the estimates are statistically higher at $29-$48 (lower income group) and $57-$237 (higher income group). These estimates can be contrasted with the €39 found in [5] for a 10% increase in effectiveness of a vaccine against a severe pandemic (€39 of 2013 is equivalent to $53 in current American dollars). Note that for the specifications not including *β*_Free_, the WTP metrics are lower, especially for the higher income group. The willingness to pay for each additional month of protection and that for each month since introduction are both of similar magnitude. For example, the RPL 2 estimate for a full year of protection is roughly $132 for the lower income group. The same group has a willingness to pay of $183 for a vaccine that has been around for a full year. Note how the models that incorporate *β*−Free, i.e. Cond. logit 2 and RPL2, produce WTP estimates that are generally larger and even more extreme for the higher income group.

In terms of who recommends the vaccine, compared to a vaccine being supported by the primary care physician PCP of the respondent, the negative WTP estimates for the media and WHO indicate distrust by the American public. CDC was as trusted as their PCP with a WTP estimate that was not significant for any of the two models for models Cond Logit 1, 2, and RPL 1. Although the estimates cannot be directly contrasted with those of [5] (due to the use of different levels), the study in the Netherlands found a WTP of €70 for a vaccine recommended by the PCP, €82 for international organizations, and €97 for the Dutch government. In the case of side effects, Dutch respondents valued in −€35 when side effects of the vaccine were not known [5]. Even though we used the same definition of side effects as [8], the design of that study did not include a monetary attribute that could be used for deriving WTP metrics.

Finally, regarding origin of the vaccine, compared to a German vaccine the lower income group is willing to pay a premium of up to $63-$157 for an American vaccine and up to $37-$112 for a British vaccine. All four models are associated with a statistically lower WTP estimate for the UK vaccine as compared to USA origin. For a Chinese vaccine, respondents would expect an average maximum compensation of $43-$74, for the lower income group. Republicans expect an even higher compensation of $108-$120 for the same income group.

### Conditional logit: opting in and out from taking the vaccine

Table 7 transforms the ***γ*** parameters into odds ratios for easier interpretation of how the covariates affect the odds of opting out from getting a COVID-19 vaccine. For example, the odds of opting out African Americans in the random parameter logit models are roughly two times higher than that for whites. Also for the random parameter logit models, the odds of not getting a COVID-19 vaccine are almost 70 times higher for those who stated that oppose to vaccination in general (5.4% of the sample). Statistical significance coincides with that of the original ***γ*** parameters, with Hispanic being the only covariate that is not significantly different from 0.

**Table 7:** Point estimates of odds ratios of opting out

Variable	Cond. logit 1	RPL 1	Cond. logit 2	RPL 2
*Opt-out: exp*(***γ***)				
Opt-out constant (mean)	2.8691	1.9581	2.2218	1.4294
Opt-out constant (st.dev.)		26.0613		26.2920
Male	0.5166	0.2397	0.5163	0.2387
Household Income [$10K]	0.9829	0.9458	0.9829	0.9456
Education BSc	0.8614	0.7130	0.8635	0.7157
Education Postgraduate	0.5084	0.2205	0.5084	0.2200
Age	1.0260	1.0587	1.0261	1.0589
Black or African American	1.3507	2.1467	1.3534	2.1522
Hispanic	0.9878	1.0455	0.9898	1.0473
Asian or Asian American	1.0869	1.4032	1.0830	1.3964
Religion extremely important	0.8490	0.6102	0.8495	0.6103
Democrat	0.5863	0.3182	0.5853	0.3161
Tested for COVID-19	0.8698	0.6958	0.8699	0.6951
Had COVID-19	0.7151	0.8176	0.7122	0.8147
No health insurance	1.2167	1.6589	1.2200	1.6672
Had flu shot	0.5974	0.3597	0.5967	0.3584
Against vaccination	5.8745	64.3117	5.8747	64.7659
Older than 65 in household	0.7882	0.6098	0.7882	0.6095
BMI	0.9905	0.9802	0.9905	0.9802
Underlying condition	0.7232	0.5049	0.7240	0.5050
Works full time	0.6585	0.3928	0.6572	0.3913

On average and ceteris paribus, individuals that are more likely to opt in for taking a COVID-19 vaccine are: males, more affluent, graduates of higher education, democrat, less fit, and full time workers. Those who by the time of the survey already had received vaccination against the seasonal flu, had been tested for COVID-19, actually had COVID-19, and those stating that religion is extremely important for them also have a direct higher likelihood of opting in for vaccination. In terms of a direct (from odds ratios that are higher than 1) higher likelihood of opting out from vaccination, we find older individuals, African Americans, individuals without health insurance, and those who – as stated above – are generally against vaccination. This latter category has the largest odds ratio of opting out.

That men are more open than women to take the vaccine has also been found in recent public opinion surveys (for example, see [18, 19],). In [18] women were reported to be 71% more likely to opt out of vaccination, followed by blacks or African Americans who were associated with a 41% higher likelihood of opting out.

### Latent class conditional logit analysis

Latent class conditional logit LCL models were fitted varying the number of classes (*Q*∈{2,3}), and explicitly accounting for the case of a free vaccine or not. An LCL model with 3 classes and including *β*_Free_ was selected for discussion, based on minimum BIC (Table 8) as decision rule [20]. Models with 4 or more classes failed to converge.

**Table 8:** LCL summary

Number of classes *Q*	*β*_Free_	Loglikelihood	BIC
2	N	-15746	32014.71
3	N	-15077	31030.82
2	Y	-15697	31935.17
3	Y	-15025	30957.67

For the selected LCL model with three classes (*Q*=3) and accounting for the vaccine being free, Table 9 summarizes the LCL point estimates.

**Table 9:** LCL point estimates

Variable	Class 1	Class 2	Class 3
Expected size	22.29%	29.01%	48.69%
*Preferences:* ***β***			
Free	0.6442	0.1848^.^	0.6492***
Cost [$]	-0.0032	-0.0074***	-0.0011***
Effectiveness [%]	0.0181**	0.0217***	0.0098***
Protection [months]	0.1457***	0.0587***	0.0228***
Incubation [days]	-0.0018	-0.0173***	-0.0016
Severe side effects [out of 10^6^]	-0.0063***	-0.0053***	-0.0020***
Mild side effects [out of 10]	-0.0829	-0.0761***	-0.0434***
Introduced [months]	0.1063	0.0463**	0.0504***
*Origin (base: Germany)*			
USA	0.6132	0.6284***	0.2927***
UK	0.4724	0.2328**	0.3436***
China	0.0906	-0.6744***	0.0478
*Recommends (base: PCP)*			
Media	-0.6104	-0.7302***	-0.5711***
CDC	-0.5464	0.0455	-0.1903**
WHO	-1.4917**	-0.1555^.^	-0.2911***
*Heterogeneity:* ***Π***			
Cost × Income ≥ $120K	0.0045	0.0005	0.0025***
Effectiveness × Wave 2	0.0073*	0.0022*	0.0013
China × Republican	-0.6470*	-0.3443***	-0.0612
*Opt out*			
Opt-out constant	5.5959***	1.6116***	-2.8583***
*Class assignment:* ***γ***			
Constant		-0.3326**	0.1184
Male		0.5586***	1.0934***
Household Income [$10K]		0.0180**	0.0398***
Education BSc		0.1750**	0.2049***
Education Postgraduate		0.4588***	1.1254***
Age		-0.0068***	-0.0408***
Black or African American		-0.0432	-0.4359***
Asian or Asian American		0.3971**	0.0423
Religion extremely important		-0.5129***	0.1798***
Democrat		0.4294***	0.8728***
Tested for COVID-19		0.4021***	0.3170***
Had COVID-19		0.9076***	0.9269***
No health insurance		-0.3106***	-0.2865***
Had flu shot		0.8060***	0.9930***
Against vaccination		-1.7113***	-2.4752***
Older than 65 in household		0.2735***	0.3519***
BMI		0.0045	0.0159***
Underlying condition		0.4914***	0.6363***
Works full time		0.0959^.^	0.6028***
Loglikelihood	-15025.00		
BIC	30957.67		

Note: Significance codes ^.^*p*<0.1,∗*p*<0.05,∗∗*p*<0.01,∗∗∗*p*<0.001

For completeness, and also because adding constraints within classes can cause convergence issues, Table 9 presents the preference parameters for each class, even when the point estimates are not significantly different from 0. Whereas point estimates of these preference parameters are derived for each of the three classes, assignment to classes – recovered in the ***γ*** parameters of the multinomial logit representing the probability of belonging to a specific class – is relative to a class that is set as reference. In our model, class assignment is relative to belonging to Class 1.

In terms of preferences, Class 1 only show statistical significance (with expected signs) for effectiveness, months of protection, number of individuals with severe side effects, the vaccine being mainly supported by the WHO, as well as an increase in the valuation of effectiveness within the second wave of data collection, and for the negative perception of a Chinese vaccine among republicans. Almost all covariates are statistically significant in Class 2, with the exception of either CDC or WHO backing up a specific vaccine, the decrease in the marginal utility of income when income increases, and the increment in the probability of choosing a vaccine when there is no out-of-pocket cost. In the case of Class 3, statistical significance is lacking for waiting time in days to develop immunity, the vaccine being Chinese (as compared to a German origin), and the eventual change in valuation of effectiveness after efficacy of actual COVID-19 vaccines were made known (i.e., during Wave 2). Note that ceteris paribus and looking at the opt-out constant, individuals in Class 1 are the most likely to opt out from taking a vaccine, followed by those in Class 2.

Looking at class assignment, Class 1 is more likely to be composed by women, less affluent, older, African American individuals who also lack health insurance and have not taken the flu shot. Class 3, with individuals that are the least likely to opt out from vaccination, is likely composed by affluent, younger, highly educated men who work full time and are also democrats. Table 10, which presents point estimates of the odds ratios of class assignment, can be used to understand likely class composition in a more straightforward manner. Note that statistical significance coincides with that of Table 9 for the parameters ***γ***, but it should be interpreted against 1 instead of against 0. In terms of the expected size of each class, as reported in Table 9, Class 2 represents 29.01% of the sample, and Class 3 is 48.69%. These shares were found using the following class assignment strategy: for each individual and using Bayes’ theorem [21], posterior probabilities of class assignment [20, 22] were derived conditional on the actual sequence of choices to the experiment (cf. [23, 24],). The posterior multinomial logit probabilities were then used to randomly assign an individual to one of the three classes.

**Table 10:** LCL point estimates of class assignment odds ratios, relative to Class 1

Variable	Class 2 vs. Class 1	Class 3 vs. Class 1
Constant	0.7171**	1.1257
Male	1.7482***	2.9844***
Household Income [$10K]	1.0182**	1.0406***
Education BSc	1.1912**	1.2274***
Education Postgraduate	1.5822***	3.0814***
Age	0.9932***	0.9600***
Black or African American	0.9932	0.6467***
Asian or Asian American	1.4875**	1.0432
Religion extremely important	0.5988***	1.1970***
Democrat	1.5363***	2.3936***
Tested for COVID-19	1.4950***	1.3730***
Had COVID-19	2.4784***	2.5267***
No health insurance	0.7330***	0.7509***
Had flu shot	2.2389***	2.6993***
Against vaccination	0.1806***	0.0841***
Older than 65 in household	1.3146***	1.4218***
BMI	1.0045	1.0160***
Underlying condition	1.6346***	1.8895***
Works full time	1.1006^.^	1.8895***

Note: Significance codes ^.^*p*<0.1,∗*p*<0.05,∗∗*p*<0.01,∗∗∗*p*<0.001

Finally, Table 11 summarizes point estimates of the maximum willingness to pay for marginal changes in the attributes of the vaccines, based on the preference estimates of Table 9. Note that Class 1 is omitted due to the marginal disutility of cost for Class 1 is not significantly different from 0 – and hence WTP estimates would be inflated.

**Table 11:** LCL maximum marginal willingness-to-pay (WTP) estimates

Variable	Class 1	Class 2	Class 3
Effectiveness [%]		$2.94	$8.55
Protection [months]		$7.96	$19.94
Incubation [days]		-$2.35	-$1.40
Severe side effects [out of 10^6^]		-$0.72	-$1.76
Mild side effects [out of 10]		-$10.32	-$37.88
Introduced [months]		$6.27	$44.02
*Origin (base: Germany)*			
USA		$85.23	$255.54
UK		$31.58	$302.38
China		-$91.47	
*Recommends (base: PCP)*			
Media		-$99.04	-$498.64
CDC			-$166.16
WHO		-$21.10	-$254.15

Whereas valuation of effectiveness, protection, incubation, and side effects in relatively similar to those reported for the conditional logit models with a continuous random effect, valuations of origin and recommender for individuals in Class 3 are notably more extreme than what was previously found.

## Discussion and conclusions

With the three leading vaccine efforts in the Western World having an initial efficacy over 90% (c.f. flue vaccine efficacy usually ranging in 40-60%), which was described as a remarkable research outcome, success of containing spread of COVID-19 has depended on rollout of vaccination programs of an unprecedented size. Using microdata collected from a choice experiment, with data collection that started before any vaccine had approval for emergency use in the US, this study has analyzed early response by Americans to hypothetical vaccines aimed at stopping pandemic spread of the SARS-CoV-2 virus. Estimates of the fitted choice models in this study are informative for current and future immunization programs.

**Fig. 3:**
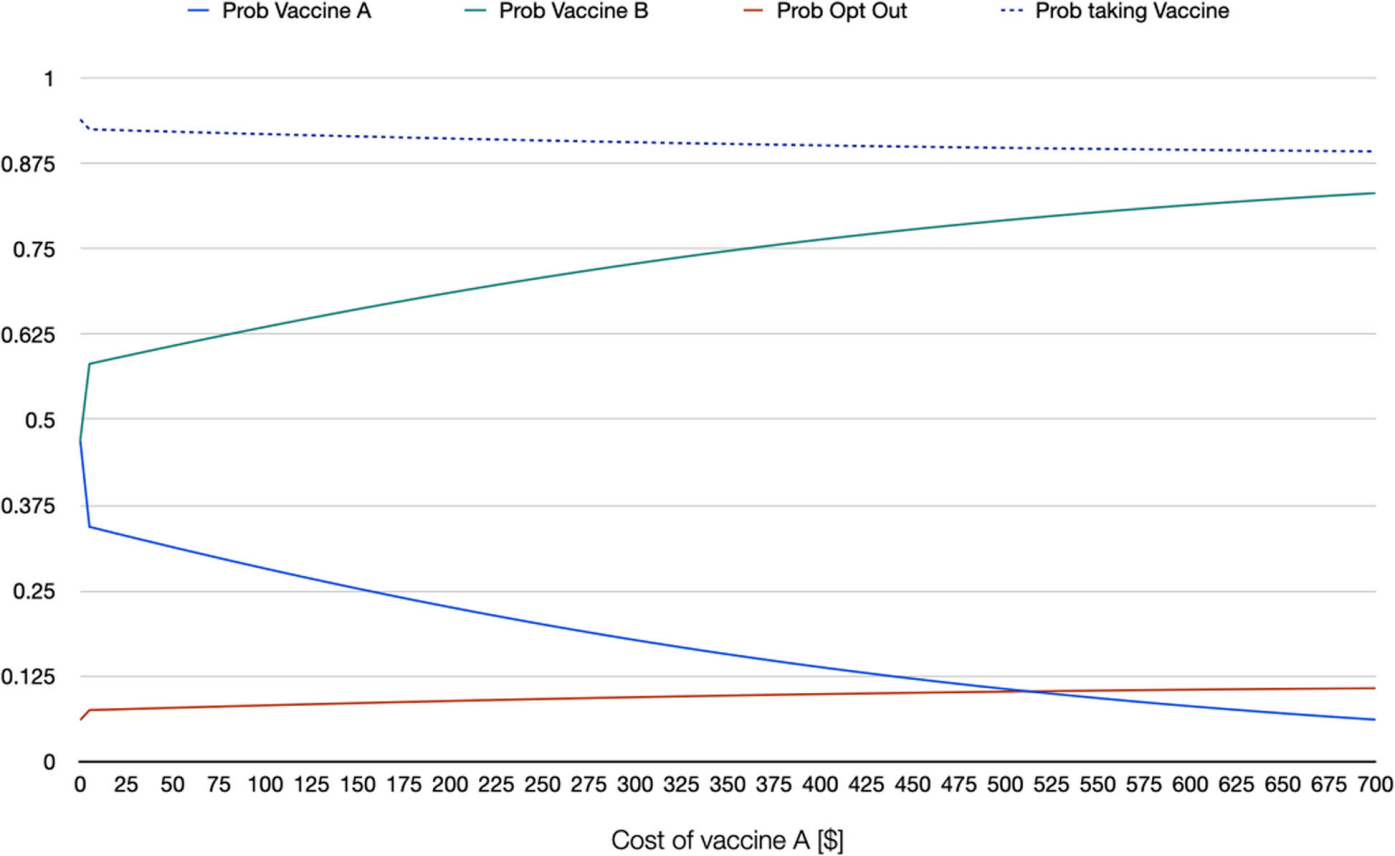
Scenario 1: RPL 2 probabilities when changing cost of vaccine A, representative individual

**Fig. 4:**
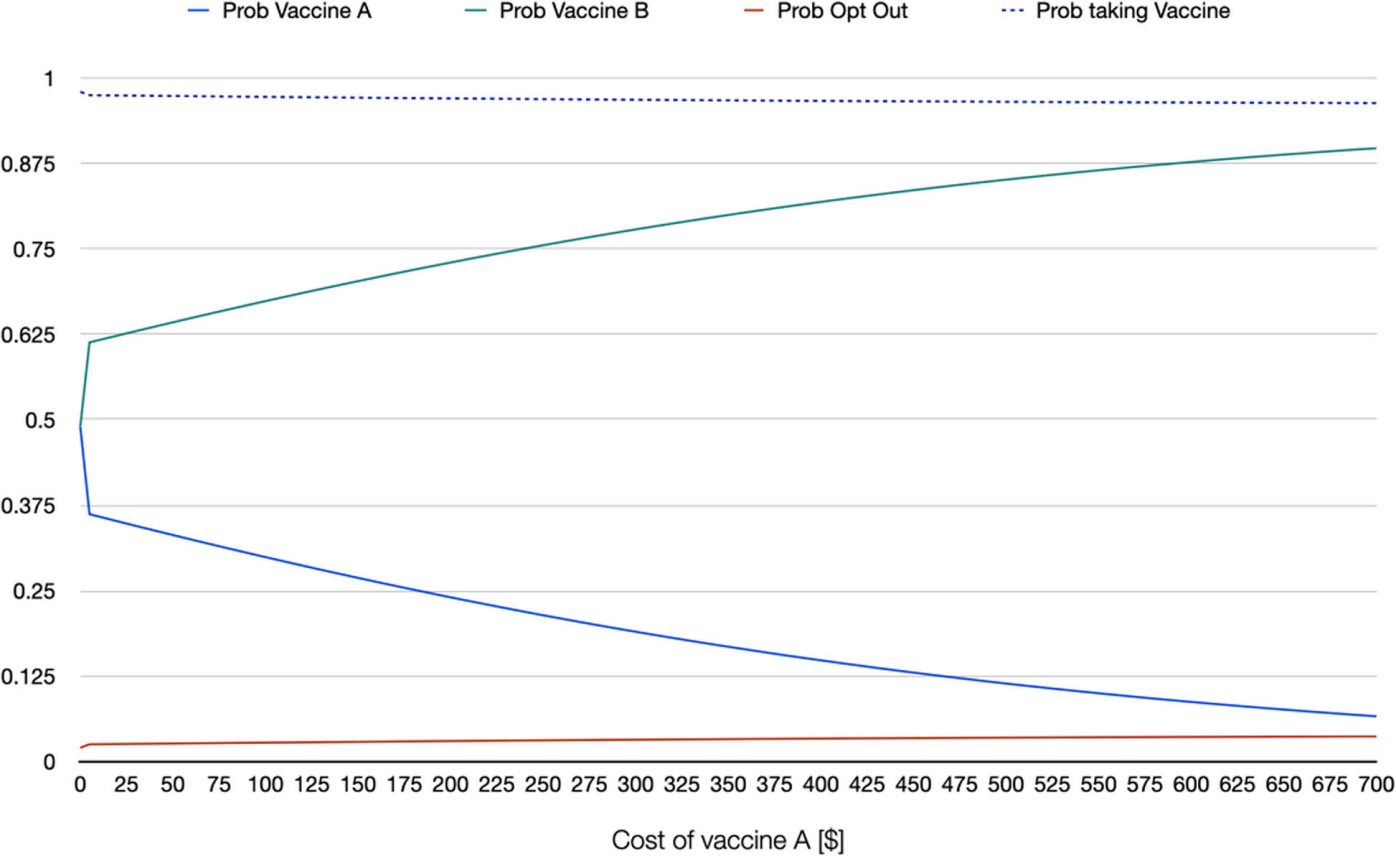
Scenario 2: RPL 2 probabilities when changing cost of vaccine A, representative individual who is democrat

**Fig. 5:**
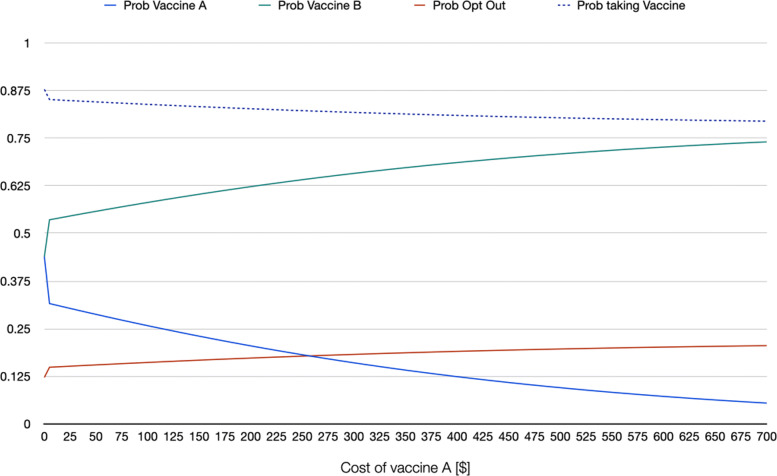
Scenario 3: RPL 2 probabilities when changing cost of vaccine A, representative individual who is African American

**Fig. 6:**
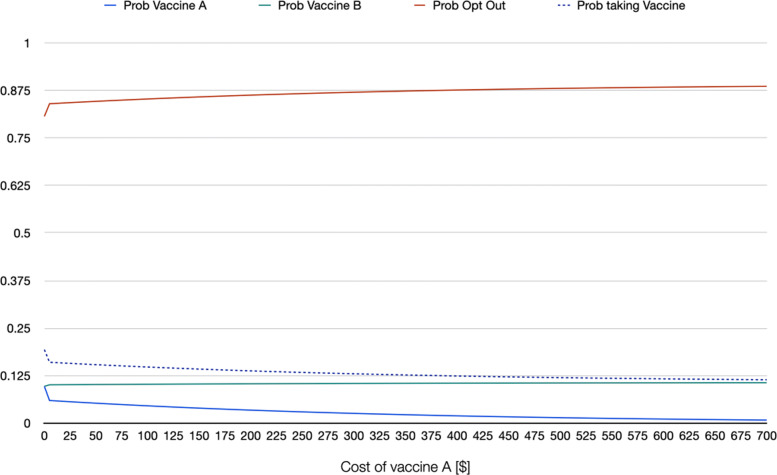
Scenario 4: RPL 2 probabilities when changing cost of vaccine A, representative individual who stated to be against vaccination in general

As example of informative scenario analysis of willingness to being vaccinated, using the estimates from a random parameter logit that accounts for nonlinearity in the marginal utility of the cost of the vaccine (RPL 2 model) the series of graphs in Appendix show the behavior of the probability of choosing each of the two hypothetical vaccines as well as that of opting out as a function of specific changes in the attributes of one of the vaccines. In the graphs, it is assumed that the following representative individual: a white male individual, aged 42 (sample mean), with a BMI of 31 (sample mean) and household income of $62,236 (sample mean) is choosing among vaccine A, vaccine B, and not taking any vaccine if A and B are the only two options. On the one hand, vaccine B in this hypothetical scenario is free, has effectiveness of 95%, it takes 21 days for antibodies to develop, offers 12 months of protection against COVID-19, 4 out of 10 people develop mild side effects, 10 out of 1,000,000 develop severe side effects, and was introduced 6 months before the moment of vaccination. These attribute levels roughly mimic what was known about the available vaccines at the beginning of 2021. On the other hand, vaccine A is identical to vaccine B with the exception of the attribute on the X-axis of each graph. For instance, in Fig. 3 in Appendix, cost of vaccine A is changed from being free up to a cost of $700. The graph shows the change in the three probabilities (of choosing vaccine A, vaccine B, and not taking any of the two available vaccines) as a function of cost of vaccine A, ceteris paribus (meaning that all other attributes of vaccine A are identical to those of vaccine B). Note how when the cost of vaccine A approaches $515, the probability of taking that vaccine and opting out are identical, whereas taking the free vaccine reaches a probability of 0.80. Fig. 4 in Appendix repeats the same exercise, but for an individual that in addition to the characteristics mentioned above is also democrat. Note how the probability of taking the vaccine is overall higher for this individual. For an individual who is black or African American (Fig. 5 in Appendix), the probability of taking the vaccine is lower even if both vaccines A and B are free (with a probability of 0.88, against 0.94 for the first representative individual, and 0.98 for the democrat representative individual). If the first representative individual stated to be against vaccination, the probability of taking the vaccine even when both alternatives are free drops to 0.19 (Fig. 6 in Appendix).

Vaccine hesitancy has been an actual problem in the US; as of February of 2022 according to CDC, 75.4% of the American population has received at least one dose, 64.2% are fully vaccinated, and only 42.4% has received a booster. 65.8% of females are fully vaccinated, whereas the figure is 61.6% for men. CDC only reports booster figures for those aged 65 and older; within that group, 72% of booster recipients were non-Hispanic white, 8% were African American, and 9% were Hispanic.

Outcomes of this study can be used to target specific segments of the population to ensure that vaccination campaigns are successful, especially by addressing vaccination gaps that emerge from the analysis and that match observed trends. These results could also become informative for future pandemics. For example, a worrying result is the modeled higher refusal of the vaccine among blacks and African Americans. In 2020 CDC reported that black or African Americans of non-Hispanic ethnicity have rate ratios of COVID-19 cases, hospitalization, and death that are respectively 1.4, 3.7, and 2.8 times higher than those for white, non-Hispanic individuals [25]. However, for blacks or African Americans, the 95% confidence interval of the odds ratio of opting out from taking the vaccine in our random parameter logit RPL 2 model is [1.35,3.35]. For the same model, the 95% confidence interval of the odds ratio of opting out for males is [0.18,0.33]. From information that 42 states are reporting, as of January of 2022, 55% of blacks or African Americans in those states have received at least one dose, whereas the figure goes up to 65% for Hispanics.

Americans are and will have access to the vaccine at no out-of-pocket cost, as indicated by CDC, but our models provide estimates of the maximum willingness to pay for marginal improvements in the attributes of the vaccine. The willingness to pay metrics show that individuals would be ready to pay substantial out-of-pocket amounts for effective and safe vaccines. Regarding the vaccine being free as opposed to having an actual out-of-pocket cost, the 95% confidence interval of the odds ratio of taking the vaccine (against opting out) in our random parameter logit RPL 2 model is [1.51,1.85].

Finally, there are limitations in our work. First, and although there is growing evidence of external validity of accuracy of the use of choice experiments, the analysis is based on stated intentions to stimuli (the attributes of the vaccines) that are hypothetical. Even within the hypothetical choice context, choice experiments do not ask individuals to directly elicit valuation of each experimental attributes but rather statistical inference on those preferences is made based on choices alone. Second, even if the choice context presented in our study does mimic actual availability of two vaccines, in practice most individuals did not a choice about which vaccine to take. Also, COVID-19 immunization is being offered for free, but we presented alternative vaccines with an out-of-pocket cost in some choice situations. Rather than being a limitation, having a cost attribute for the vaccines serves two purposes: having estimates of private valuation of the vaccine attributes that could be used for evaluating welfare-improving health interventions, and providing evidence of preferences of future scenarios in which vaccines would be expected to be paid for. These scenarios include eventual future pandemics, or immunization in the future once the pandemic is controlled but the SARS-CoV-2 virus still would be around. Future research should look into evolution of intentions to receive the vaccine and contrast those intentions with revealed preferences from actual behavior observations after vaccines were made available to the general population.

## Appendix A: Scenario graphs

## Data Availability

Not applicable.
